# Consequences of 22q11.2 Microdeletion on the Genome, Individual and Population Levels

**DOI:** 10.3390/genes11090977

**Published:** 2020-08-22

**Authors:** Małgorzata Karbarz

**Affiliations:** Institute of Biology and Biotechnology, University of Rzeszow, Pigonia 1, 35-310 Rzeszow, Poland; karbarz.m@gmail.com

**Keywords:** 22q11.2 microdeletion, 22q11.2 deletion syndrome, consequence, penetrance

## Abstract

Chromosomal 22q11.2 deletion syndrome (22q11.2DS) (ORPHA: 567) caused by microdeletion in chromosome 22 is the most common chromosomal microdeletion disorder in humans. Despite the same change on the genome level, like in the case of monozygotic twins, phenotypes are expressed differently in 22q11.2 deletion individuals. The rest of the genome, as well as epigenome and environmental factors, are not without influence on the variability of phenotypes. The penetrance seems to be more genotype specific than deleted locus specific. The transcript levels of deleted genes are not usually reduced by 50% as assumed due to haploinsufficiency. 22q11.2DS is often an undiagnosed condition, as each patient may have a different set out of 180 possible clinical manifestations. Diverse dysmorphic traits are present in patients from different ethnicities, which makes diagnosis even more difficult. 22q11.2 deletion syndrome serves as an example of a genetic syndrome that is not easy to manage at all stages: diagnosis, consulting and dealing with.

## 1. Introduction

Chromosomal 22q11.2 deletion syndrome is the most common chromosomal microdeletion disorder occurring in approximately 1 in every 1000 fetuses [1,2], affecting 1 in every 4000 individuals [3,4,5,6]. De novo deletion, that is, neither parent has the deletion, is currently identified in 90–95% patients. Microdeletions are approximately 0.7–3 million base pairs in size and result in varied clinical symptoms [7,8,9]. The 22q11.2 region is one of the most structurally complex areas in the human genome mainly due to several large blocks of LCRs (low copy repeats) or segmental duplications [10]. These LCRs are above 96% identical, which is why the locus is susceptible to meiotic errors [11,12,13]. Like in other microdeletion and microduplication syndromes, the 22q11.2 region can be deleted or duplicated [14]. Clinical manifestations of 22q11.2 duplication syndrome (Dup(22)(q11)) can be different even in members of the same family. These include: hypotonia, slow growth, delay in development, intellectual disability, heart defects, and velopharyngeal insufficiency [15,16]. The incidence of Dup(22)(q11) is estimated as being half of that of 22q11.2DS [15]. Theoretically, meiotic errors that lead to duplication and deletion should happen at equal frequencies; however, in early selection during gametogenesis, one is chosen more often than the other. In the case of Dup(22)(q11), many individuals have little or no symptoms, and the duplication may not be identified. This coincides with a general observation that microduplications seem to cause milder or no clinical manifestations compared with reciprocal microdeletion [14]. The penetrance of 22q11.2 microdeletion is high, meaning that almost all individuals with the deletion will have some of symptoms. Clinical presentations of 22q11.2DS can be associated the dysfunction of many organs such as, among others: the heart, palate, brain, immune systems, and endocrine, genitourinary, gastrointestinal systems [17,18,19,20,21,22]. Clinical symptoms are so varied that, in the absence of typical craniofacial traits and other common birth defects, such as those of the heart or palate, a diagnosis may be difficult to make. Some adults are only diagnosed after the birth of a sick child [23]. The genetic mechanism of phenotypic variations of the quantity and intensity of these presentations cannot be explained by deletion size or by 22q11.2 hemizygosity. Apart from multi-gene, a combination of other factors, such as modifications of protein coding and regulatory genes outside the 22q11.2 region as well as the sensitivity of individual genes within the 22q11.2 region to gene dosage and alleles in the counterpart chromosome, may influence the 22q11.2DS phenotype [18]. Even members of the same family with identical genetic alterations are characterised by high phenotypic diversity, variable expression and incomplete penetrance of some of the traits [23,24]. The possible association between the clinical phenotype and the size and location of 22q11.2 deletion may be masked by other genetic and/or epigenetic modifying factors [25]. The correlation between genotype and phenotype is difficult to determine, due to great inter- and intrafamilial clinical diversity, even between mono-oval twins [23,26].

## 2. Genome Level

### 2.1. Genotype-Specific Penetrance

Chromosome 22, where the 22q11.2 microdeletion occurs, is approximately 50 Mbp in length, and the 0.7–3 Mbp deletion represents 1.4–6% of the chromosome and 0.023–0.1% of the whole genome. A copy-number variation (CNV) is a difference in the genome due to deletion or duplication of large regions of DNA on some chromosomes Using variability in the copy number, it is calculated that the human-to-human genetic variance is at least 0.5% (99.5% similarity) [27,28]. According to recent papers, there are approximately 4400–7500 structural variations (SVs) per genome, which is more than twice the number of variants per genome detected by the 1000 Genomes Project [29,30,31]. From a statistical point of view, the 22q11.2 microdeletion genome is no different as we all have some mutations, deletions, or duplications; although, sometimes, we are just lucky that they are not in critical regions or do not manifest themselves phenotypically. 22q11.2 microdeletion affects 1 in every 4000 individuals—0.025% of a population of 7.5 billion. Collins et al. estimated that 0.13% of individuals may carry SV that meet the standards for clinically significant incidental findings. Very large SVs (>1 Mb) were present in 3.9% of samples (14,237 genomes) [31].

Although little phenotypic information has been provided by the participants of the 1000 Genomes Projects and other recent projects, the study donors are non-vulnerable adults capable of contributing to the project, who are thus likely to lack any clear phenotype of serious disease. The participants pass all quality thresholds and represent the general adult population [31]. It is possible that the penetrance of disease alleles and genotypes may be much smaller and more complex than previously thought. Conventionally, most studies of human inherited disease have identified a disease genotype based on clinical phenotype [32]. This strategy is different from identifying a phenotype based on the genotype. The former approach eliminates the entire question of penetrance because it focuses solely on those individuals among whom the mutation in question was widespread. It follows that a given variant may be truly causative in a group of people with a specific disease and may also be present in healthy people. In certain cases, straight correlation lines cannot be drawn from recognised genotypes to particular clinical phenotypes, because there are many people who possess a mutation/genotype associated with a disorder but do not show other characteristics of the disorder or may even be asymptomatic. This decreased penetrance may not be the rule, but it is far from an unusual exception. Lupski et al. (2011) claim that, ‘for a given individual, what is important to know is not only the number and location of pathogenic variants taken one at a time, but also the unique composition of his or her genome-wide mutational burden.’ It appears that certain mutations alone are insufficient to cause disease, which occurs in the presence of certain genetic variations, either allelic or non-allelic, as well as facilitative environmental factors. Penetrance is better understood as a genotype-specific rather than gene-specific or disease-specific phenomenon. However, it is worth emphasizing that 22q11.2 microdeletion syndrome involves the deletion of multiple genes in combinatorial complexity created by four LCRs. Phenotypic diversity is expected to be more variable than in the case of a single deleterious variant and depends on many factors, such as the kind of mutations on the other allele, epistatic mutations elsewhere in the genome, and genome-wide mutation burden. There is no simple correlation between genotype and phenotype, and this should be taken into consideration in the diagnosis of 22q11.2 deletion syndrome and many other diseases.

### 2.2. Microdeletion and Surroundings

**Figure 1:**
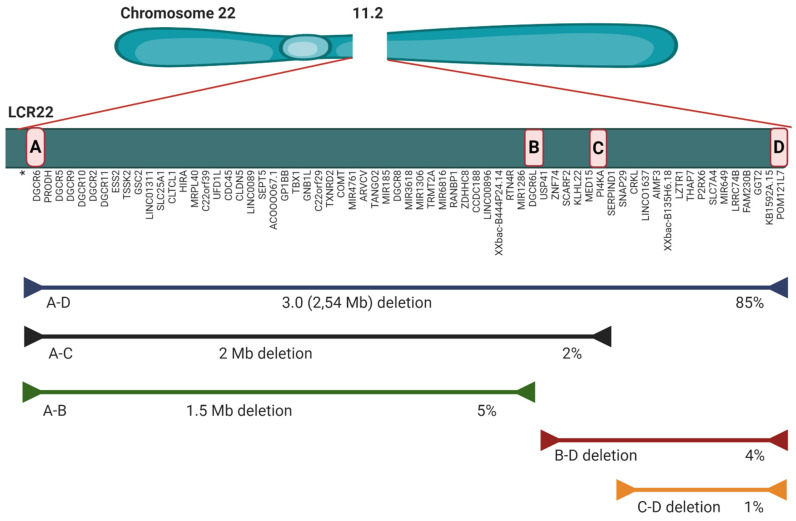
Scheme of the 22q11.2 microdeletion locus [4,7,18,33]. * Pseudogenes and undefined transcripts are not included in the figure.

**Figure 2:**
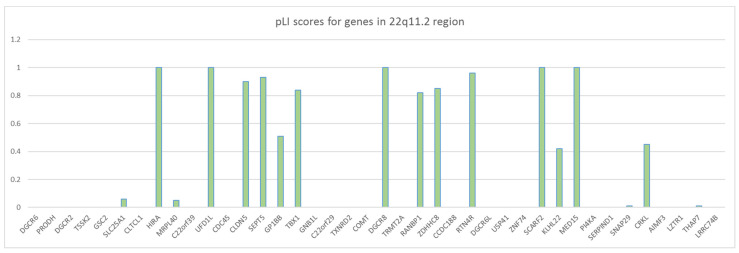
pLI scores for genes in the 22q11.2 region. The arrangement of genes according to their order in the 22q11.2 region. pLI score values were obtained from the gnomAD browser [37].

The microdeletion in 85–90% of individuals is 2.54 Mb (historically known as 3 Mb) (A to D), which results in hemizygosity in approximately 106 genes: 46 protein coding genes, 24 pseudogenes, 7 microRNAs, 12 long noncoding RNAs, 2 small nuclear RNA and additional undefined transcripts (Figure 1) [4,33]. The rarer deletion (5–8%) is 1.5 Mb (A to B) and leads to haploinsufficency in 30 coding genes, 6 micro RNAs, and 9 long non-coding RNAs. There are also three more rarely occurring deletions: B-D (4%), A-C (2%) and C-D (1%) (Figure 1). All types of microdeletion are caused by incorrect rearrangement of chromosomes during meiosis. These exchanges involve eight large, paralogous LRC22s (A-H) or segmental duplications that are located along the 22q11.2 region. The location of the breakpoint within an LCR does not have a huge impact on the 22q11.2DS phenotype [34]. Optical mapping showed the difference between LCR22 structures in normal and deletion-containing haplotypes. Specific NAHR (non-allelic homologous recombination) locations and a genomic signature associated with the deletion were detected. After an analysis of the potential deletion breakpoints in 30 22q11.2DS families, the results show that, for a group of 88 people, favored recombination happens between FAM230 gene members and segmental duplication orientations within LCR22A and LCR22D [35]. Well-characterized genes that play a role in the 22q11.2 phenotype are among others: T-Box 1 Transcription Factor (TBX1), DiGeorge Syndrome Critical Region 8 (DGCR8), Catechol-O-Methyl Transferase (COMT), Septin 5 (SEP5), Proline Dehydrogenase (PRODH), Zinc-finger DHHC—type Containing 8 (ZDHHC8), Crk-like Adaptor Protein L (CRKL). pLI scores were designed to show the probability that a given gene is extremely intolerant of loss-of-function (LoF) [36]. The closer the pLI is to one, the more LoF intolerant the gene appears to be (pLI ≥ 0.9 points to an extremely LoF intolerant gene). Available pLI scores for genes in the 22q11.2 region were obtained from data in the gnomAD browser (Figure 2) [37]. Although TBX1 has a high pLI score (0.84), its—and other genes’—haploinsufficiency does not explain the penetrance and clinical phenotypes’ severity. This situation can be observed even between members of the same family with the same TBX1 mutations [18]. TBX1 regulates the expression of about 2000 [38]. This gene plays role in the patterning of the pharyngeal arches and pouches [4,5,38]; however, its haploinsufficiency does not lead to an expected level of malformations. There are other genetic and epigenetic factors, within and outside chromosome 22q11.2 that change the clinical phenotypes of 22q11.2DS [38,39]. TBX1 interacts with the KMT2-familly and the TBX1-KMT2 complex controls by chromatin marking the low-level expression of thousands of genes. TBX1 also interacts with the SWI-SNF-like BAF complex (the ATP-dependent chromatin remodelling complex ) to help the remodeling of chromatin. It follows that the levels of TBX1 may modulate thousands of transcript expressions. TBX1 is downregulated by another, also deleted, gene—DGCR6; so, fluctuations in DGCR6 may affect TBX1 levels. The transcript levels of DGCR6 should be reduced to 50% due to haploinsufficiency, but some individuals actually have higher levels of DGCR6 than normal controls [40]. Another key gene is DGCR8. This gene is necessary for miRNA biogenesis. DGCR8 binds to primary miRNA (pri-miRNA) transcripts with DROSHA, which cleaves pre-miRNA into precursor miRNA (pre-miRNA). The expression of hundreds of miRNA is modulated due to reduction in DGCR8 expression, caused by 22q11.2DS. Micro RNA deregulation is observed in 22q11.2DS patients (Table 1.) [41,42]. Recent research reports that protoporphyrins can increase miRNA biogenesis in DGCR8 haploinsufficient in in vitro mouse cells, so this could be potentially therapeutic for 22q11.2DS patients [43]. Other fragments embedded in the deleted segment that could affect the clinical presentations are long noncoding RNAs (lncRNA) and small nuclear RNAs (snoRNAs). Noncoding RNAs and miRNA deregulation impact embryo development and immune, cardiac, and neurological functions postnatally. There are also differences in other parts of the individual’s 22q11.2DS genome, outside the affected locus [44]. Rare deleterious SNP (single nucleotide polymorphism) screening detected polymorphism in some genes in 22q11.2DS patients with CHD (congenital heart disease) [45]. GLUT3 duplication, located on chromosome 12p13.3, can also occur, and is only pathogenic in combination with 22q11.2DS [46].

Taken together, the haploinsufficiency of coding genes and their epigenetic regulation, diverse noncoding RNA, miRNA deregulation and genetic differences external to affected locus may influence disease severity and penetrance in 22q11.2DS individuals, but exactly how this happens remains an open question.

### 2.3. Mouse Model of the 22q11.2 Deletion Syndrome

Several mouse models have been developed based on the conserved linkage between human chromosome 22 and mouse chromosome 16 [47,48,49,50]. The most recent imitates 3.0 Mb deletion (Del(3.0 Mb)/) and was generated using the CRISPR/Cas9 system. The deletion was introduced between Pi4ka and Hira genes on mouse chromosome 16. CGH (comparative genomic hybridization) was used to confirm the reduction in genomic copy numbers in this region. Adult male mutants were evaluated with the use of: behavioural tests (prepulse inhibition, fear-conditioning memory, measurements of locomotor activity, visual discrimination learning), circadian behavioural rhythm, and visual-evoked potential. Mice with the deletion (Del(3.0 Mb)/+) were hypoactive—they travelled shorter distances and were less active in their subjective night. This may reflect the tendency of 22q11.2DS patients to tire more quickly. When it comes to social interaction tests, mice with the deletion had encountered difficulties with social recognition of a novel mouse, while the social memory of Del(3.0 Mb)/+ mice was intact. Additionally, Del(3.0 Mb)/+ mice showed a reduction in auditory prepulse inhibition and attenuated cue-dependent fear memory. Del(3.0 Mb)/+ mice displayed a quicker adaptation to experimental jet lag as compared with wild-type mice. This model is the first model with 3.0 Mb deletion and could be very useful for understanding psychiatric disorders in 22q11.2DS [51].

## 3. Individual Level

### 3.1. 22q11.2DS Phenotype

22q11.2 deletion syndrome affects many organs with different severity and penetrance (Table 2). Palatal anomalies are very common in 22q11.2DS patients. These are mainly: velopharyngeal insufficiency (27–92%), submucous cleft palate (5–16%), cleft palate (9–11%), and bifid uvula (5%) [5]. In terms of immune profile, immunodeficiency is very common. Sixty-seven percent of patients experience impaired T cell production, six percent—an IgA deficit, 23 percent—humoral defects (relevant in reaction to vaccines). The immune system usually normalises itself by the first year of life; however, more infections may occur in adulthood [23,52,53]. Cardiac anomalies in 22q11.2 individuals include: Tetralogy of Fallot (20–45%), an interrupted aortic arch (5–20%), ventriculoseptal defects (10–50%), truncus arteriosus (5–10%) [5,17,54] During the screening of patients with congenital heart disease, 22q11.2DS is detected in half of the cases of interrupted aortic arch, among one third of the patients with truncus arteriosus, and in one-sixth of patients with Tetralogy of Fallot. These studies demonstrate that there is a reasonable amount of cases of 22q11.2 deletion among patients with these conditions [55,56]. Endocrine problems include mainly: hypocalcemia (50%) and growth hormone deficiency (4%). Hypocalcemia is often resolved at a neonatal stage. Calcium deficiency may recur in times of disease and stress. Calcium levels should be monitored yearly. Major findings in renal abnormalities include having a single kidney (12%), multicystic dysplatic kidney 4%, or hydronephrosis 5% [57]. Feeding and swallowing anomalies include: gastroesophageal reflux, esophageal dysmotility, constipation, prolonged tube feedings, and G-tube placement [58,59]. Tortuous retinal vessels (58%) and posterior embryotoxon (69%) are common ophthalmologic abnormalities. Neurological problems include: cerebral atrophy (1%) and cerebral hypoplasia (0.4%). Skeletal abnormalities are not very common. They include cervical spine anomalies (40–50%), vertebral anomalies (19%) and anomalies of lower limbs (15%) [5]. Additionally, there are characteristic dysmorphic features, which are mild and are not visible for non-professionals or can only be noticed when individuals with 22q11.2DS are gathered together. They are the following: elongated face, low-set small dysplastic ears, microstomia, small teeth, congenital tooth or enamel agenesis, almond-shaped eyes, hypertelorism, a prominent long bulbous nose, retro- and micrognathia, a short neck and characteristic arachnodactyly [60]. According to human phenotype ontology database Abnormal facial shape (HP:0001999), Epicanthus (HP:0000286), Bulbous nose (HP:0000414), Wide nasal bridge (HP:0000431), Prominent nasal bridge (HP:0000426), Telecanthus (HP:0000506), Upslanted palpebral fissure (HP:0000582), and Low-set ears (HP:0000369) are very frequent [61]. Psychiatric problems occur mainly in adolescence. About 25% of 22q11.2 individuals have schizophrenia [62,63,64,65]. One per 100 patients with schizophrenia has 22q11.2 deletion [5]. The beneficial effects of omega-3 supplementation on attentional control and in transition to psychosis could support its early use in the 22q11DS population [66]. Pituitary dysmaturation is also present and could be associated with pleiotropic psychopathology and atypical neurodevelopment [67]. Sleep problems and motor coordination problems are also common in young 22q11.2DS patients [68]. Development delays need to be checked at every step of infancy and childhood, as early intervention can help provide support for children with the deletion [69,70]. The most common problems are motor delays and speech difficulty, which can be connected with very frequent conductive hearing impairment (HP:0000405) and muscular hypotonia (HP:0001252) [61,71,72,73,74]. Delays in reaching motor milestones and the emergence of language are common in children with 22q11.2DS. Motor delays may be associated with congenital heart disease and are less severe, while delays in language development are more noticeable and are not associated with any major medical issues [75]. A recent pilot study of motor phenotypes shows that the developmental history of 22q11.2DS children differs from that of their siblings (control). They fail to thrive (42%), are more likely to experience feeding difficulties (84%), and parents have reported on their clumsiness (79%). Only 32% are able to talk by the age of 2, in contrast to 92% of their siblings. Sixty-eight percent stated special educational needs and were using a health care plan. Children with 22q11.2 deletion syndrome are able to button their clothes at 6.2 years (median) and to do up their laces at 9.75. Upon examination, 95% show evidence of movement disorders and dystonia [71]. The mean IQ of such individuals is about 70, and 22q11.2DS children have problems with mathematics and other skills that require abstract reasoning [76,77,78,79]. Children with 22q11.2 deletion are also withdrawn and struggle in social situations, which makes their school lives harder [80,81,82,83,84]. Parents of children with 22q1.2 deletion syndrome are more stressed compared to parents of typically developing children, so this could also be another burden on the young 22q11.2DS patient [85]. Such children nevertheless usually go through the normal educational system, with the help of their parents and teachers [86]. It is not easy for 22q11.2 to gain employment after graduation; however, about 33% of 22q11.2DS adults were employed in an open market and about 25% in an assisted-employment environment [87]. Professions occupied by 22q11.2 adults include: cooks, farmers, security guards, maintenance staff, office employees, nurses, homemakers, early childhood educators, family therapists [23]. The life expectancy for adults with 22q11.2DS is lower than expected among other members of their families. According to data on 309 adults with 22q11.2DS, the range of deaths is 18.1–68.6 years, with a median age of 46.4 [88].

### 3.2. Diagnosis

For families in which the 22q11.2DS has been identified, either in one of the parents and/or in child preimplantation genetic diagnosis (PGD) and prenatal diagnosis (PND) are available [89,90]. Another indicator for diagnosis is a demonstration that a parent carries a balanced or unbalanced chromosome rearrangement involving 22q11.2. PGD aims at the selection of an embryo without 22q11.2 deletion by analysis of cells taken from oocytes, zygotes or embryos produced by in vitro fertilization (IVF), and then transporting them into the womb. The biopsied cells are analysed by the FISH (fluorescent in situ hybridization) method [91]. The first PGD by FISH for 22q11.2DS was reported almost twenty years ago, but currently it is very rarely used [92]. Prenatal diagnosis is available by non-invasive methods: foetal ultrasonography and echocardiography, and invasive methods: chorionic villus sampling (CVS) or amniocentesis followed by FISH (fluorescent in situ hybridization), GCH or SNP-array [4,93]. It is possible to evaluate fetal cells collected through amniocentesis, typically performed at 15–18 weeks gestation, or through CVS at approximately 10–12 weeks gestation. In addition to these methods, cell-free foetal DNA testing—a non-invasive method that enables the sequencing of the foetal DNA obtained from maternal plasma—is under investigation [94,95]. Sequencing analysis of ccfDNA (circulating cell-free DNA) has been shown to enable accurate prenatal detection of foetal aneuploidies, including trisomies of chromosomes 21, 18, and 13. There are trials which extend these analyses to examine subchromosomal copy number variants through the sequencing of ccfDNA [94,95]. Currently, screening for the 22q11.2 deletion in individuals from the general population is considered when anatomic abnormalities, for example, CHD and/or other associated abnormalities, such as cleft palate, polydactyly, diaphragmatic hernia, renal anomalies or polyhydramnios, are identified on foetal ultrasonography, or when a foetus is considered high risk following non-invasive prenatal screening [96]. In the case of high-risk pregnancy, a high-resolution ultrasonic test for palatal and other related abnormalities, or an echocardiography for cardiac abnormalities, may enable assessment between 18 and 22 weeks of gestation. Recently used methods such as an SNP (single nucleotide polymorphism microarrays) can diagnose a foetus with 22q11.2DS with or without prenatally recognisable features.

22q11.2 deletion syndrome is postnatally diagnosed through SNP array, multiplex ligation-dependent probe amplification (MLPA), chromosomal microarray (CMA) or less frequently used FISH. When a new born has many of the 22q11.2DS features, the SNP array is recommended to confirm the diagnosis, as about 5–10% of individuals with a clinical finding of the 22q11.2 deletion syndrome have normal routine cytogenetic studies and normal FISH results [4,97,98]. These rare patients are diagnosed with DiGeorge syndrome [4]. SNP array detects copy number variations in any part of the genomes and is the preferred initial test for 22q11.2DS. CMA is an older method, and it identifies substantial gains or losses of chromosomal regions in any part of the genome. MLPA is also an older method, which identifies gains or losses of chromosomal regions in up to 45 areas within the genome and uses seven probes within the typical 22q11.2 deletion region. FISH checks whether a specific section on one chromosome contains deletions, duplications, or translocations, and can be used for detecting mosaicism, screening family members, and prenatal diagnosis [99]. All parents of children with 22q11.2DS should be screened in order to find individuals that are slightly affected and those with a low level of somatic mosaicism. If the parents of an individual with 22q11.2DS have normal tests, the risk that they will have another baby with the deletion is low, yet greater than that of the general population since parents have been identified with germline mosaicism or low-level somatic mosaicism [100]. 22q11.2DS is inherited in an autosomal-dominant way. Around 93 percent of probands have de novo deletions, while 7 percent inherit a parent’s 22q11.2 deletion. The children of affected individuals have a 50 percent risk of inheriting the 22q11.2 deletion. For couples in which one partner has the 22q11.2, prenatal genetic counselling includes discussions about preimplantation genetic diagnosis, using IVF and/or the above-mentioned prenatal non-invasive and invasive diagnosis methods. Additionally, if the cffDNA tests are accepted for clinical use, then pre-test and post-test counselling will be necessary, as apart from foetal diagnosis an unexpected diagnosis of maternal deletions or duplications in the 22q11.2 region may be obtained [94,95]. Of great importance during genetic counselling, no matter whether during pregnancy, postnatal or in adolescence, is emphasising that the phenotype is unpredicted and the overall function of the patient depends on many genetic and environmental factors. It is important to note that variable CNV penetrance is likely to be explained by the influence of the rest of the genome, at least in part. These findings have important implications both for genetic counselling and for the understanding of variable penetrance associated with genetic variants that have a large phenotypic impact. Knowledge of the deleterious impact of a genetic variant enriched in information concerning the parental phenotype could make it possible to successfully predict the developmental domains that are most likely to be severely affected. [101].

## 4. Population Level

### 4.1. 22q11.2DS in Different Populations

22q11.2 deletion syndrome is an underdiagnosed condition. Many patients are diagnosed secondary to CHD (75%). Physical examinations differ between population groups, making the 22q11.2DS diagnosis difficult, especially for individuals of African descent, as they have different craniofacial dysmorphisms compared to the standard recognised anomalies found in Caucasians [102]. Only learning problems and ear anomalies are present to the same extent across ethnicities. To help with diagnosis in countries where laboratory tests are limited or unavailable, researchers have created a website where all the facial, hand and foot dysmorphy are presented on photographs for different counties [103]. They have also proposed digital facial technology as an alternative tool to molecular testing among mixed populations. This could be really a helpful tool for 22q11.2 deletion syndrome diagnosis.

Data for the 22q11.2 population can be obtained from the Decipher database, which uses Ensembl Resources [104]. According to the Decipher database (214 matching patients for 22q11.2 deletion, 109 46XX, 105 46XY), the origin of the deletion is de novo in 24%, of unknown origin in 48%, maternally inherited in 19%, paternally inherited in 4%, and a result of imbalance arising from balanced parental rearrangement in 5%. The five most common phenotypes are: intellectual disability (81 patients), micrognathia (67), hypocalcemia (64), ventricular septal defect (64), abnormality of the pinna (58). In female patients, the deletion is de novo in 23%, of unknown origin in 50%, maternally inherited in 20%, paternally inherited in 3% and a result of imbalance arising from balanced parental rearrangement in 5%. The five most common phenotypes are: intellectual disability (39), arachnodactyly (35), micrognathia (32), ventricular septal defect (32), abnormality of the pinna (31). In male patients, the deletion is de novo in 26%, of unknown origin in 46%, maternally inherited in 20%, paternally inherited in 3%, and a result of imbalance arising from balanced parental rearrangement in 5%. The five most common phenotypes are: intellectual disability (42), hypocalcemia (37), micrognathia (35), ventricular septal defect (32), delayed speech and language development (28). The prevalence of specific phenotypes appears to be similar in both genders, except for delayed speech and language development, which is three times higher in boys than in girls. However, this is most likely due to the higher occurrence of communication, language, and speech disorders in boys than in girls in the general population and may not be related to the 22q11.2 microdeletion [105].

### 4.2. Dealing with the Syndrome

Once diagnosed, if the symptoms are severe then the 22q11.2DS requires a well-established care plan. As 22q11.2 microdeletions have different expressivity, each patient requires an individualised, multidisciplinary and coordinated care plan. This kind of solution has already been introduced in Canada, where there is a special hospital unit dedicated to 22q11.2DS patients [106].These kind of centres or programs in hospitals are also provided in a few states in the USA. In Europe there are clinics which do not have special programmes but offer their service on the site The International 22q11.2 Foundation [107]. In countries where there are special centres, the parents need to find their own way to deal with the syndrome and to find specialists for their children [108]. Some helpful materials could provide guidelines [3,109].

Couples where at least one of the potential parents has the 22q11.2 deletion should visit a genetics counsellor. Pregnancies should be monitored more carefully as, especially when the mother has 22q11.2, more problems may occur as a consequence of the deletion. The mother may have, for example, a calcium deficiency, hypothyroidism or heart problems, in which case the application of an interdisciplinary approach increases the chances of a safe and successful pregnancy, childbirth and first weeks of life [110]. Published in 2015, the first set of guidelines for adults addressed the management of neuropsychiatric, cardiovascular, endocrine, reproductive and psychosocial issues as well as genetic counselling, proposing practical approaches to their health issues and their identification, evaluation, monitoring and management [3].

The 22q11.2 microdeletion has incomplete penetrance in humans and the severity of disease depends on the complete genetic make-up in concert with environmental factors [111,112,113]. The individual genetic code is lifetime stable, but genes can be silenced or over-expressed. Epigenetics may respond to environmental factors and influence genome expression. In rats, different programming of the GR (glucocorticoid receptor) exon 17 promoter in the hippocampus of offspring of high and low LG (licking/grooming) maternal care was observed. The differences that emerge between day 1 and 8 after birth remain stable thereafter. These differences include epigenetic changes such as histone acetylation, DNA methylation, and the occupancy of the promoter with the transcription factor [114]. A comprehensive analysis of the hippocampus transcriptome of the adult offspring of high and low LG maternal care revealed differences in a few hundred genes. This suggests a wide change in epigenetic programming in the brain of the offspring, as a consequence of maternal care. Recognition that not only is the genome programmed by the epigenome, but that this programming may be equally important to genome functionality as the genetic sequence, opens up possibilities for new approaches to the nature of gene–environment interaction. Recent data from behavioural studies have helped develop current understandings of the relationship between social environment and epigenetic programming, as well as the lifelong dynamic nature of the epigenome. Behaviour and the epigenome have a bilateral influence on one another, with behaviour having implications on epigenetic programming and epigenetic programming affecting behaviour [115]. In the case of 22q11.2DS, awareness of this relationship is extremely important. The baby and mother, whenever possible, should be together all the time, especially babies with health problems. The results show that the mother–child relationship influences the 22q11.2 child’s overall functioning. Relationships between children with 22q11.2DS and their parents tend to be more difficult [116]. Issues with a child’s social engagement can be non-intrusively alleviated through dyadic therapy [116]. Another environmental factor that can influence the functioning of children is socioeconomic status (SES). A higher SES of children with 22q11DS is correlated with better overall functioning, social skills and less frequent oppositional defiant behaviour [117]. However, for the control subjects, SES is correlated with cognition and achievement rather than behaviour, which shows that the behavioural phenotype of children with 22q11DS is influenced by psychosocial factors related to methods of upbringing and schooling as well as the epigenetic regulation of gene expression. While delayed development is greater in hereditary cases, the reasons for this may be socioeconomic, not genetic [117]. Genetic counselling should include recognition of family socioeconomic status and if needed, help should be offered. Parent’s intellectual capacities are another genetic-environmental factor. The research shows a correlation between the effect of the 22q11.2 deletion and the parents’ level of education, especially in the case of verbal IQ. This may be explained by an appeal to environmental factors related to education level. Nevertheless, intelligence is strongly hereditary, and therefore the same genetic factors may influence the IQ of a child and of their parent. This is also true in cases of de novo 22q11.2 deletion, as the data shows [118].

## 5. Summary

In 22q11DS, a lot depends on the genome, epigenome, parental IQ level, socioeconomic status and parental–child relation. To some extent, parents do have an influence on their child’s emotions and intellectual level by stimulation during early childhood, for example: early reading, counting techniques, physical, social activities and most important hugging and acceptance of the situation. Extra psychological therapy if needed is also a great option.

When it comes to the research on 22q11.2DS this should be focused on finding the parts of the genome/epigenome that influence the phenotypic expression of the syndrome and help parents and patients themselves to modulate their life by normal behaviours, such as exercise and diet, as all these factors influence our life. Despite the difficulties it entails, reduced penetrance can aid the identification of drugs, environmental factors, and methods of intervention that limit the penetrance of a given pathological variant. The continuum of human genetic variation spans neutral polymorphisms, functional polymorphisms and variants of disease susceptibility, and pathological mutations with high penetrance. Epigenetic and environmental factors influence the impact of disease phenotypes. As a result, phenotype contributions are highly individualised.

It is important to look at the genome as a whole and interpret genetic test results within their wider background, such as epigenetics and the environment. The case of 22q11.2DS is a great example of an unpredicted phenotype, as, despite great genetic knowledge and an excellent genetic test, we are not able to say why monozygotic twins with exactly the same deletion have different phenotypes. The way the genome deals with this change is more important than the change itself, and the way we as a society deal with the genetic test result is also much more important than the result itself.

## Figures and Tables

**Table 1:** MicroRNA expression dysregulation in 22q11.2 patients and mouse model [41,42]. miRNA located on 22q.11.2 locus in bold.

miRNA:	Expression Level:	Tissue/Organ:	Possible Contribution to:
miR-185	0.4 times lower (H) lower (MM) different expression (H) *: CHD+/− hypocalcemia+/− low CD3 T cell count +/−	Synapses (H), hippocampus (MM)	Immune system deficiency, neurological abnormalities, hypotonia
miR-194	lower (H, MM) different expression: CHD+/− hypocalcemia+/− low CD3 T cell count +/−	prefrontal cortex (MM), hippocampus (MM)	dendric and spine development deficits in hippocampus, acute myocardial infraction
miR-208	higher(H)	no data	cardiac disease
miR-190	higher(H)	no data	cardiac disease
miR-1	higher (H)	no data	
miR-150	lower (H) different expression: CHD+/− low CD3 T cell count +/−	no data	low number of mature T and B cells
miR-363	lower (H)	no data	no data
miR-15b-3p, miR-324-5p, miR-720	different expression: CHD+/− hypocalcemia+/− low CD3 T cell count +/−	no data	no data
miR-363, miR-23b	different expression: CHD+/−	no data	no data
miR-21	different expression: hypocalcemia+/− low CD3 T cell count +/−	no data	no data
miR-145	different expression: hypocalcemia+/−	no data	no data
miR-365-5p	different expression: CHD+/− low CD3 T cell count +/−	no data	no data
miR-29a	different expression: low CD3 T cell count +/−	no data	no data

* H—human, patient with 22q11.2DS, MM—mouse model, +/− patients with compared to patients without: CHD, hypocalcemia, low CD3 T cell count.

**Table 2:** Selected clinical manifestation in patients with chromosome 22q11.2 deletion syndrome [5,23,61].

Clinical Manifestation	Frequency in 22q11.2 DS	Human Phenotype Ontology Database *
Palatal anomalies	69–100%	Cleft palate (HP:0000175), Abnormality of the pharynx (HP:0000600), Platybasia (HP:0002691)
Learning disabilities	>95%	
Speech delay	79–84%	
Cardiac anomalies	49–83%	Abnormality of cardiovascular system morphology (HP:0030680), Abnormal aortic arch morphology (HP:0012303), Truncus arteriosus (HP:0001660), Ventricular septal defect (HP:0001629), Abnormal pulmonary valve morphology (HP:0001641), Tetralogy of Fallot (HP:0001636), Atrial septal defect HP:0001631
Immunodeficiency	77%	Immunodeficiency (HP:0002721), Abnormality of the tonsils (HP:0100765), Hypocalcemia (HP:0002901), Impaired T cell function (HP:0005435)
Developmental delay in infancy	75%	
Ophthalmologic abnormalities	7–70%	Posterior embryotoxon (HP:0000627), Corneal neovascularization (HP:0011496), Ptosis (HP:0000508)
Endocrine	60%	Hypoplasia of the thymus (HP:0000778), Hypoparathyroidism (HP:0000829)
Behaviour/psychiatric problems	9–50%	
Developmental delay in childhood	45%	Short stature (HP:0004322)
Renal anomalies	36–37%	Renal hypoplasia (HP:0000089)
Feeding and Swallowing Problems	35%	Anorectal anomaly (HP:0012732), Constipation (HP:0002019)
Skeletal abnormalities	17–19%	Arachnodactyly (HP:0001166), Abnormal skull morphology (HP:0000929), Short neck (HP:0000470)
Neurologic	8%	
Dental: Delayed eruption, Enamel hypoplasia	2.5%	Abnormality of the dentition (HP:0000164), Carious teeth (HP:0000670)

* only annotations indicated as “frequent” and “very frequent” were chosen.
